# A vesicle-aggregation-assembly approach to highly ordered mesoporous γ-alumina microspheres with shifted double-diamond networks†
†Electronic supplementary information (ESI) available. See DOI: 10.1039/c8sc02967a


**DOI:** 10.1039/c8sc02967a

**Published:** 2018-08-17

**Authors:** Yang Liu, Wei Teng, Gang Chen, Zaiwang Zhao, Wei Zhang, Biao Kong, Wael N. Hozzein, Areej Abdulkareem Al-Khalaf, Yonghui Deng, Dongyuan Zhao

**Affiliations:** a Department of Chemistry , Laboratory of Advanced Materials , Shanghai Key Laboratory of Molecular Catalysis and Innovative Materials and iChEM , Fudan University , Shanghai 200433 , P. R. China . Email: dyzhao@fudan.edu.cn ; Fax: +86-21-5163-0307; b State Key Laboratory for Pollution Control , School of Environmental Science and Engineering , Tongji University , Shanghai 200092 , P. R. China; c School of Physical Science and Technology , ShanghaiTech University , Shanghai 201210 , P. R. China; d Bioproducts Research Chair , Zoology Department , College of Science , King Saud University , Riyadh 11451 , Saudi Arabia; e Botany and Microbiology Department , Faculty of Science , Beni-Suef University , Beni-Suef , Egypt; f Biology Department , College of Sciences , Princess Nourah bint Abdulrahman University , Riyadh , Saudi Arabia; g State Key Laboratory of Transducer Technology , Shanghai Institute of Microsystem and Information Technology , Chinese Academy of Sciences , Shanghai 200050 , P. R. China

## Abstract

Alumina materials have widely been used in industrial fields, such as catalysis and adsorption. However, due to the fast sol–gel process and complicated crystalline-phase transformation, the synthesis of alumina materials with both highly ordered mesostructures and crystallized frameworks remains a great challenge. Herein, we report a novel vesicle-aggregation-assembly strategy to prepare highly ordered mesoporous γ-alumina microspheres with unique shifted double-diamond networks for the first time, by using diblock copolymer poly(ethylene oxide)-*b*-poly(methyl methacrylate) (PEO-*b*-PMMA) as a template and aluminum isopropoxide as a precursor in a tetrahydrofuran (THF)/hydrochloric acid binary solvent. During the gradual evaporation of THF and H_2_O, the as-made Al^3+^-based gel/PEO-*b*-PMMA composites can be obtained through a co-assembly process based on the hydrogen bonding interaction between hydroxyl groups of alumina oligomers and PEO segments of the diblock copolymers. The formed composites exhibit a spherical morphology with a wide size distribution (diameter size 1–12 μm). Furthermore, these composite microspheres possess an inverse bicontinuous cubic mesostructure (double diamond, *Pn*3[combining macron]*m*) with Al^3+^-based gel buried in the PEO-*b*-PMMA matrix in the form of two intertwined but disconnected networks. After a simple calcination at 900 °C in air, the structure of the resultant mesoporous alumina changes to a relatively low symmetry (shifted double diamond, *Fd*3[combining macron]*m*), ascribed to the shifting of the two alumina networks due to loss of the templates. Meanwhile, the unit cell size of the alumina mesostructure decreases from ∼131 to ∼95 nm. The obtained ordered mesoporous alumina products retain the spherical morphology and possess ultra-large mesopores (∼72.8 nm), columnar frameworks composed of γ-alumina nanocrystalline particles (crystal size of ∼15 nm) and high thermal stability (up to 900 °C). As a support of Au nanoparticles, the formed Au/mesoporous γ-alumina composite catalysts have been used in the catalytic reduction of 4-nitrophenol with a high kinetic constant *k* of 0.0888 min^–1^, implying promising potential as a catalyst support.

## Abstract 2

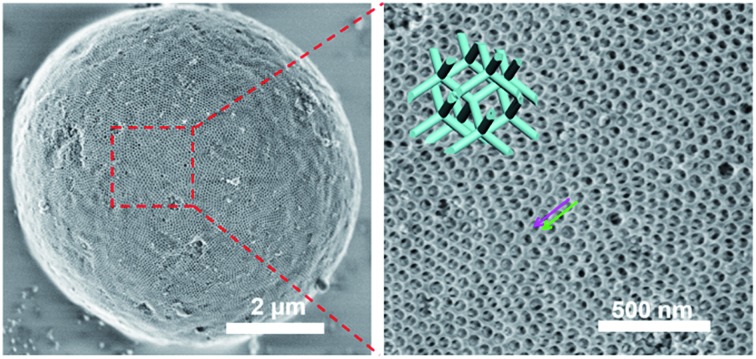
Highly ordered mesoporous γ-alumina microspheres with a shifted double-diamond mesostructure have been synthesized *via* a vesicle-aggregation-assembly approach.

## Introduction

Porous alumina materials have received considerable attention owing to their wide applications in catalysis and adsorption realms,^1^–^8^ the performance of which is strongly affected by their porosity, microstructures and crystalline phases. Enormous efforts have been devoted to the structural manipulation of mesoporous alumina,^9^–^14^ ascribed to the superior features of ordered mesostructures, such as the relatively large pore size, high pore volume and low diffusion resistance. Nevertheless, different from the controllable sol–gel chemistry of silicates,^15^–^19^ the fast hydrolysis–condensation reactions of alumina precursors always result in phase separation or undesirable co-assembly with templates. On the other hand, the metastable transition phases of alumina also have a great influence on their performance. Especially, γ-phase alumina possesses various excellent properties (*e.g.*, hardness, corrosion resistance, hydrolytic stability, amphoteric character and thermal stability), making it a promising candidate for further applications.^20^ However, to obtain alumina materials with the γ-phase, high-temperature treatment is usually inevitable, which always results in the destruction of the formed morphology and mesostructure. Therefore, it is highly desirable but rather difficult to fabricate mesoporous alumina materials with both highly ordered mesostructures and γ-phase frameworks simultaneously.

Until now, several strategies have been performed to prepare such materials, including the nano-casting route and surfactant-directing route. For the former, ordered mesoporous carbons, owing to predesigned ordered mesostructures, rigid frameworks and easy selective removal,^21^,^22^ are usually adopted as a hard template to synthesize ordered mesoporous γ-aluminas. With ordered mesoporous carbon CMK-3 as a rigid template, Zhang and co-workers have prepared ordered mesoporous γ-alumina by repetitive filling and hydrolysis processes and a subsequent stepwise calcination procedure.^23^ By functionalizing the ordered mesoporous carbon with nitric acid and controlling the infiltration times, Wu *et al.* demonstrated an effective approach to obtain ordered mesoporous γ-alumina replicas with different pore architectures.^24^ Nevertheless, these synthetic routes are laborious and time-consuming, which are poorly suitable for massive production. In contrast, the surfactant-directing route to prepare ordered mesoporous materials is more feasible and economical. However, for the alumina precursors, the susceptibility to hydrolysis and the tendency to crystallize at a high temperature always result in the formation of disordered mesostructured aluminas,^25^–^27^ giving rise to great trouble in the controlled synthesis of ordered mesoporous γ-aluminas. Until now, only few studies on the synthesis of such materials have been reported with this approach.^8^,^10^,^11^,^28^ By using AlCl_3_·6H_2_O as a precursor and the block copolymer poly(ethylene-*co*-butylene)-*b*-poly(ethylene oxide) (KLE) as a soft template, Kuemmel *et al.* have reported the synthesis of ordered mesoporous γ-alumina through a dip-coating approach.^10^ A detailed research on the preparation of mesoporous γ-alumina was performed by Yan and co-workers with organic alumina as precursors and ethylene oxide-based surfactants as templates in the presence of additives such as nitric or citric acid.^11^ Despite these successes, some drawbacks, such as the strict control of relative humidity, the assistance of chelants, the use of expensive surfactants and the adoption of complicated synthetic procedures, are always encountered in the soft template-based synthesis. Furthermore, pore sizes of these mesoporous alumina materials are all less than 20 nm, impeding effective mass transportation especially in macromolecule-involved applications.

Recently, taking full advantage of the interlocking property of the bicontinuous mesostructures (double gyroid,^17^,^29^–^37^ double diamond^38^–^42^ and double primitive^43^–^49^), Che and co-workers have demonstrated a novel approach to prepare ordered inverse materials (silica and titania scaffolds) with ultra-large mesopores (pore size > 50 nm) by using lab-synthesized block copolymers as templates through a multilayer core–shell bicontinuous microphase-templating route.^39^,^41^,^43^ However, the morphology control of these novel mesoporous materials has not been achieved yet. Furthermore, this method was not successful to prepare mesoporous alumina analogues. Therefore, the synthesis of such ultra-large pore mesoporous γ-alumina materials with a highly ordered mesostructure and a well-defined morphology simultaneously is still a great challenge and has not been reported yet.

Herein, for the first time, we report a facile repeatable synthesis of highly ordered and ultra-large pore mesoporous γ-alumina microspheres with a shifted double-diamond mesostructure *via* a new vesicle-aggregation-assembly approach. With aluminum isopropoxide as a source and the amphiphilic diblock copolymer poly(ethylene oxide)-*b*-poly(methyl methacrylate) (PEO-*b*-PMMA) as a soft template, the as-made Al^3+^-based gel/PEO-*b*-PMMA composite microspheres can be obtained *via* the hydrogen bonding interaction and co-assembly induced by the evaporation of the acidic tetrahydrofuran (THF)/H_2_O mixed solvents. These composite microspheres possess a diameter size of 1–12 μm and a unique inverse bicontinuous mesostructure (double diamond, *Pn*3[combining macron]*m*). After a direct calcination at 900 °C in air, the composite microspheres can be transformed into mesoporous γ-alumina microspheres with retained morphology, although the shrinkage in the unit cell size is about 27.5%. Meanwhile, the mesostructural symmetry changes to a low shifted inverse bicontinuous mesostructure (shifted double diamond, *Fd*3[combining macron]*m*) owing to the leaning of the two intertwined but disconnected networks. The highly ordered mesoporous γ-alumina materials exhibit ultra-large mesopores (∼72.8 nm), bicontinuous columnar frameworks and high thermal stability (as high as 900 °C). It is remarkable that the mesoporous frameworks are composed of fully crystallized γ-alumina nanoparticles with an average size of ∼15 nm. The ordered mesoporous γ-alumina materials can be employed as a support of Au nanoparticles, and the formed Au/mesoporous γ-alumina composites show excellent performance in the catalytic reduction of 4-nitrophenol.

## Experimental section

### Synthesis of ordered mesoporous aluminas

The diblock copolymer poly(ethylene oxide)-*block*-poly(methyl methacrylate) (PEO-*b*-PMMA) was prepared using an atom transfer radical polymerization (ATRP) method. The structural formula was calculated to be PEO_113_-*b*-PMMA_335_ according to the ^1^H nuclear magnetic resonance (^1^H NMR) spectra, and the polydispersity index (PDI) was 1.31 based on gel permeation chromatography (GPC) tests (Fig. S1, ESI†). The detailed synthetic steps and characterizations are shown in the ESI.†

The ordered mesoporous alumina samples were synthesized *via* a solvent evaporation induced vesicle-aggregation-assembly approach. In a typical procedure, aluminum isopropoxide (400 mg) was added into a THF solution (15 mL) containing PEO-*b*-PMMA (80 mg) with stirring for 30 min. Sequentially, concentrated hydrochloric acid (2.0 mL) was added into the above solution, followed by further stirring for 30 min. The obtained clear solution was poured into a Petri dish with a diameter of 15 cm to evaporate the reaction solvents at room temperature for 48 h, and further dried at 40 °C for 48 h in an oven. The as-made Al^3+^-based gel/PEO-*b*-PMMA composites were scrapped and collected. The composites were calcined in air with a ramp of 1 °C min^–1^ to 400 °C for 4 h, followed by a ramp of 10 °C min^–1^ to a certain temperature (500–900 °C) for 1 h.

The procedure of Au-loading on mesoporous γ-alumina and catalytic reduction of 4-nitrophenol were carried out according to previous reports^50^,^51^ and are shown in the ESI.†

### Measurements and characterization

Field-emission scanning electron microscopy (FESEM) images were taken using a Hitachi S4800 scanning electron microscope. Samples were directly dispersed onto conductive tapes attached on a sample holder for observation under vacuum. Transmission electron microscopy (TEM) experiments were conducted on a JEOL JEM-2100F microscope (Japan) operated at 200 kV. The samples for TEM measurements were suspended in ethanol and dropped onto Cu grids. To investigate the interior structures of the resultant mesoporous alumina microspheres, samples were embedded in a resin and cut into thin sections with a thickness of ∼100 nm in an ultramicrotome. Small-angle X-ray scattering (SAXS) measurements were carried out on a Xenocs XeUss 2.0 small-angle X-ray scattering system. Nitrogen sorption isotherms were measured at 77 K with a Micrometrics Tristar 2420 analyzer. Before measurements, all samples were degassed under vacuum at 180 °C for 6 h. The Brunauer–Emmett–Teller (BET) method was utilized to calculate the specific surface areas using adsorption data in a relative pressure range from 0.075 to 0.225. Using the Barrett–Joyner–Halenda (BJH) model, the pore volumes and pore-size distributions were derived from the adsorption branches of isotherms, and the total pore volumes were estimated from the adsorbed amount at a relative pressure *P*/*P*_0_ of 0.995. Wide-angle X-ray diffraction (XRD) measurements were conducted on a Bruker D8 Advance X-ray diffractometer using a CuKα radiation source (*λ* = 1.5406 Å). ^27^Al MAS NMR experiments were performed on a Bruker 400WB AVANCE III spectrometer with 4 mm ZrO_2_ rotors, spun at 12 kHz. Single excitation pulse experiments were performed with a 10° pulse width of 0.33 μs, an acquisition time of 10 ms and a relaxation delay of 0.3 s. The chemical shifts were referenced to 1.0 M AlCl_3_ solution. The UV-vis spectra were recorded on a PerkinElmer Lambda 750S UV-vis spectrometer at 25 °C.

## Results and discussion

**Fig. 1:**
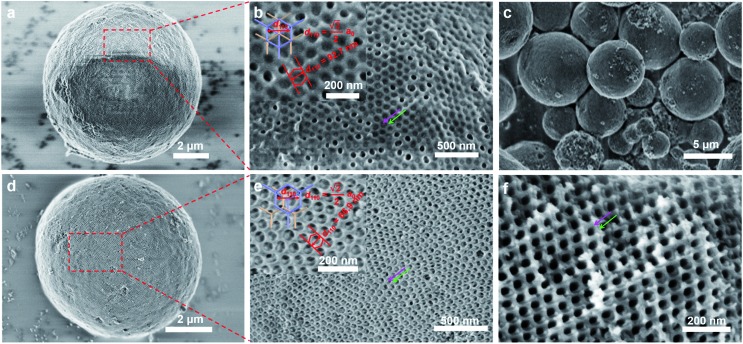
FESEM images of (a, b) the as-made Al^3+^-based gel/PEO-*b*-PMMA composite microsphere prepared by the vesicle-aggregation-assembly approach, (c–e) ordered mesoporous alumina microspheres and (f) its fragment obtained after calcination at 900 °C in air. The insets in (b) and (e) show enlarged SEM images and corresponding theoretical stick models, respectively.

FESEM images show that the as-made Al^3+^-based gel/PEO-*b*-PMMA composites prepared from the vesicle-aggregation-assembly approach are sphere-like particles with a wide size distribution (diameter size 1–12 μm) (Fig. S2, ESI†). High-magnification SEM images of a single microsphere reveal that the surface of the as-made microsphere is composed of obvious crystal facets (Fig. 1a and b). Furthermore, two intertwined but disconnected nanorod networks embedded in a matrix can be clearly observed (Fig. 1b, marked by purple and green arrows), implying that a bicontinuous cubic mesostructure is formed. The shortest circuit of the ordered mesostructure is composed of six points to form a regular hexagon (Fig. 1b inset), indicating a bicontinuous double-diamond mesostructure (space group *Pn*3[combining macron]*m*) based on Wells' theory.^52^ The unit cell size is calculated to be ∼131 nm from the *d*-spacing value (*d*_110_) of the double-diamond mesostructure. After a direct calcination at 900 °C in air, both the spherical morphology and ordered mesostructure are well retained (Fig. 1c–e), indicating that the obtained mesoporous alumina microspheres have a high thermal stability. The shifting of two intertwined but disconnected alumina networks occurs (Fig. 1e, marked by purple and green arrows), suggesting that the mesostructural symmetry changes low from *Pn*3[combining macron]*m* (double diamond) to *Fd*3[combining macron]*m* (shifted double diamond or single diamond).^33^,^42^ In addition, the unit cell size of the mesoporous alumina microspheres reduces to ∼95 nm, which is much smaller than that (∼131 nm) of the as-made Al^3+^-based gel/PEO-*b*-PMMA composites, indicating a large shrinkage (∼27.5%) of alumina frameworks due to the crystalline-phase transformation and further condensation. More importantly, it can be clearly observed that the interior of the microspheres possesses a highly ordered mesostructure (Fig. 1f), which is also composed of two shifted alumina networks. Therefore, these results clearly indicate that the whole alumina microspheres are composed of such intertwined but disconnected scaffolds with a uniform mesostructure.

**Fig. 2:**
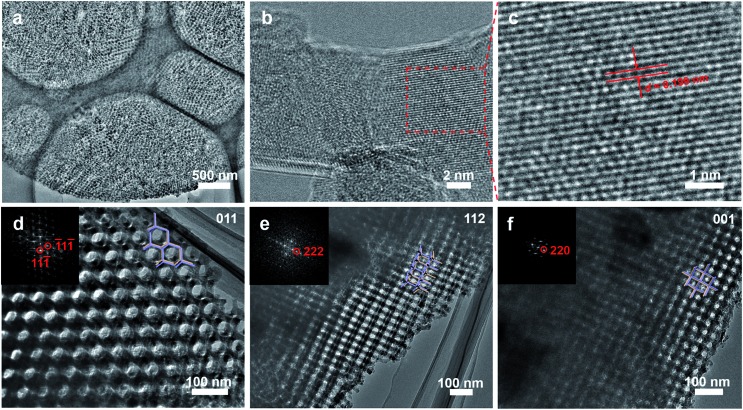
TEM images of the highly ordered mesoporous alumina prepared by the vesicle-aggregation-assembly approach after simple calcination at 900 °C in air: (a) the image of a microsection with a thickness of ∼100 nm; (b, c) high-resolution TEM images with different magnifications; (d–f) images viewed along [011], [112] and [001] directions. The insets in (d–f) are the corresponding simulated fast Fourier transform (FFT) images.

TEM images of a microsection show that the mesoporous alumina microsphere prepared by the vesicle-aggregation-assembly approach after calcination at 900 °C in air is composed of many irregular but ordered domains (Fig. 2a), suggesting that the formation of microspheres undergoes an aggregating process during the solvent evaporation. The TEM images and corresponding simulated fast Fourier transform (FFT) images of the fragments from mesoporous alumina microspheres show a characteristic shifted double-diamond mesostructure viewed from [011], [112] and [001] directions (Fig. 2d–f), respectively. According to the diffraction points, the unit cell size is calculated to be ∼97 nm, in agreement with that (∼95 nm) obtained from SEM results. In addition, high-resolution TEM (HRTEM) images of the alumina frameworks obviously show a lattice spacing of 0.199 nm corresponding to the *d*_400_ of γ-alumina (Fig. 2b and c), clearly indicating that the mesoporous alumina microspheres are composed of highly crystallized γ-alumina networks.

**Fig. 3:**
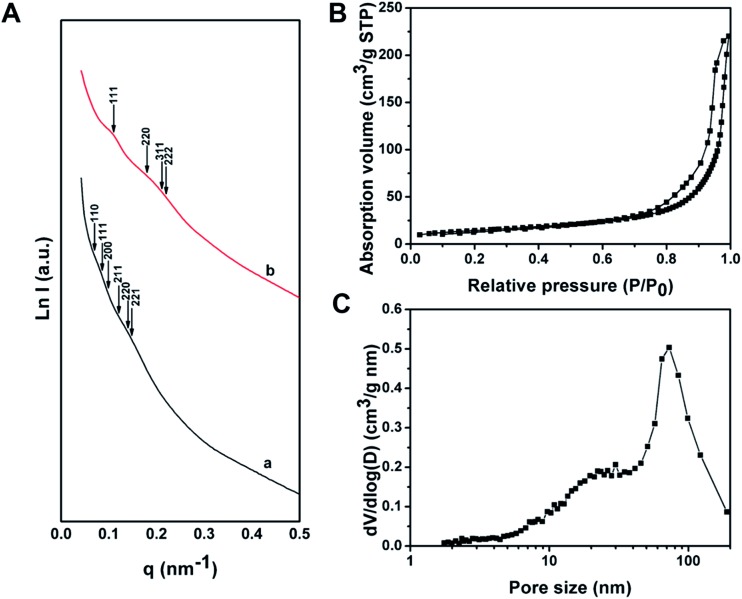
(A) SAXS patterns of (a) as-made Al^3+^-based gel/PEO-*b*-PMMA composites prepared by the vesicle-aggregation-assembly approach and (b) ordered mesoporous alumina obtained after calcination at 900 °C in air. (B) Nitrogen sorption isotherms and (C) pore size distribution of the ordered mesoporous alumina prepared by the vesicle-aggregation-assembly approach after calcination at 900 °C in air.

The SAXS pattern of the as-made Al^3+^-based gel/PEO-*b*-PMMA composites can be assigned to the possible reflections of *Pn*3[combining macron]*m* symmetry (double diamond) (Fig. 3A(a)), while the intensity of these peaks is too weak to strictly prove the attribution of the structural symmetry. The broadened and weak SAXS peaks should be attributed to the slight distortion of the formed mesostructure in each ordered domain, which inevitably occurs during the microphase separation process in the confined space. After calcination at 900 °C in air, the scattering peaks of the mesoporous alumina appear at different locations (Fig. 3A(b)), suggesting that a change of structural symmetry occurs after the removal of the amphiphilic block polymer templates. Furthermore, the allowed reflections of the *Fd*3[combining macron]*m* symmetry (single diamond), with a unit cell size of ∼99 nm based on the first 111 reflection, are consistent with the SAXS pattern of the obtained mesoporous γ-alumina. Therefore, these results further confirm a structural change to the *Fd*3[combining macron]*m* symmetry during the calcination, matching well with the SEM and TEM results.

Nitrogen sorption isotherms of the ordered mesoporous alumina microspheres prepared by the vesicle-aggregation-assembly approach after calcination at 900 °C in air exhibit representative type IV curves with a sharp capillary condensation step in a relative pressure range of 0.95–0.99 (Fig. 3B), suggesting an ultra-large mesopore. The hysteresis loop indicates high permeability between mesopores. The BET surface area and pore volume are calculated to be as low as 52 m^2^ g^–1^ and 0.34 cm^3^ g^–1^. The relative low surface area should mainly be attributed to the undetectable contribution of micropore surface area, because almost no micropore is left in the highly crystallized columnar frameworks after the high temperature calcination process. The pore size distribution derived from the adsorption branch reveals a pore size distribution at around 72.8 nm (Fig. 3C), which corresponds to the porous space created by the intertwined but disconnected scaffolds.

Thermogravimetric analysis (TGA) experiments were employed to elucidate the transformation process from the as-made Al^3+^-based gel/PEO-*b*-PMMA composites to the mesoporous γ-alumina microspheres. Four obvious weight loss intervals can be observed (Fig. S3b, ESI†): (i) a preliminary weight loss of ∼10% below 110 °C; (ii) an intense weight loss of ∼30% between 110 and 160 °C; (iii) a slow weight loss of ∼20% between 160 and 350 °C; (iv) a further loss of ∼10% until 400 °C, then an almost stable weight until 900 °C. The first two weight loss intervals below 160 °C are attributed to massive loss of H_2_O and chlorine compounds in Al^3+^-based gels with the increase of temperature (the removal of chlorine compounds is determined according to the following XRD results).^53^,^54^ The third weight loss between 160 and 350 °C occurs at the same interval as that of the template PEO-*b*-PMMA (Fig. S3a, ESI†), indicating the decomposition of the block copolymer. Finally, a further weight loss between 350 and 900 °C can be associated with the removal of H_2_O during the condensation and crystalline-phase transformation of the alumina.^54^

**Fig. 4:**
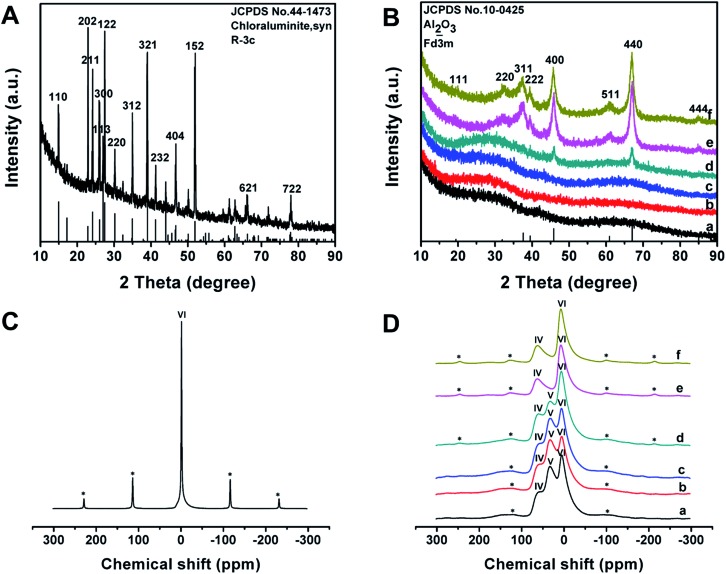
Wide-angle XRD patterns of (A) as-made Al^3+^-based gel/PEO-*b*-PMMA composites prepared by the vesicle-aggregation-assembly approach and (B) ordered mesoporous aluminas obtained after calcination at (a) 400 °C, (b) 500 °C, (c) 600 °C, (d) 700 °C, (e) 800 °C and (f) 900 °C in air. ^27^Al-MAS NMR spectra of (C) as-made Al^3+^-based gel/PEO-*b*-PMMA composites prepared by the vesicle-aggregation-assembly approach and (D) ordered mesoporous aluminas obtained after calcination at (a) 400 °C, (b) 500 °C, (c) 600 °C, (d) 700 °C, (e) 800 °C and (f) 900 °C in air. The peaks marked with a * label in (C) and (D) are spinning sidebands.

The wide-angle XRD pattern of the as-made Al^3+^-based gel/PEO-*b*-PMMA composites shows many well-resolved peaks (Fig. 4A), which can be indexed to the crystalline structure of synthetic chloraluminite (JCPDS no. 44-1473). It can be seen that the relative intensities of diffraction peaks between the composites and synthetic chloraluminite are somewhat different, implying different contents of Al^3+^-based compounds in the Al^3+^-based gels. After the temperature reaches 100 °C, the relative intensities of these diffraction peaks are completely different from that of the as-made Al^3+^-based gel/PEO-*b*-PMMA composites (Fig. S4A, ESI†), indicating that the contents of the components in the Al^3+^-based gels change greatly with increase of the temperature. Subsequently, no diffraction peak can be observed at 150 °C (Fig. S4B(b), ESI†), suggesting a rearrangement of the Al phase, which is in accordance with the intensive weight loss during this interval in the TGA curve. Afterwards, the frameworks are still composed of amorphous alumina after the removal of the block copolymer template at 400 °C (Fig. 4B(a)), then begin to crystallize at 700 °C (Fig. 4B(d)), and fully transform into the γ-alumina phase at 900 °C (Fig. 4B(f), JCPDS no. 10-0425). The average crystal size is calculated to be about 15 nm using Scherrer's equation. In addition, the elemental mapping image, together with the energy dispersive X-ray (EDX) spectrum, shows that Cl element is uniformly dispersed at a high content (up to 26.83 wt%) in the as-made Al^3+^-based gel/PEO-*b*-PMMA composites (Fig. S5, ESI†), further demonstrating that the Al^3+^-based gels are composed of various Cl-rich compounds. After calcination at 900 °C in air, no Cl element is detected in the EDX spectra of the obtained mesoporous alumina, and the atom ratio of Al to O is close to 2 : 3 (Fig. S6, ESI†), suggesting a transformation of the frameworks to Al_2_O_3_.

^27^Al-MAS NMR spectra of the as-made Al^3+^-based gel/PEO-*b*-PMMA composites reveal one resonance signal close to 0 ppm (Fig. 4C), suggesting that Al^3+^ ions mainly exist in a 6-fold coordinated form. After calcination at 400 °C to remove the template, three bands at around 63, 33, and 7 ppm are observed (Fig. 4D(a)), which can be assigned to 4- (AlO_4_), 5- (AlO_5_), and 6-fold (AlO_6_) coordination, respectively.^55^ As the temperature increases, the resonance band for 5-fold coordinated Al^3+^ ions gradually weakens and completely disappears at 900 °C (Fig. 4D), which is related to a transformation of the crystalline phase from amorphous alumina to γ-alumina, agreeing well with the results from the wide-angle XRD measurements.

An intermediate state of the reaction mixture after evaporation for 2 h was captured (Fig. S7, ESI†). Because the evaporation rate of the solvent THF is much faster than that of the zeotropic solvent water, the mixed solvent at this stage is a water-rich solvent. It can be seen that the reaction solution is opaque after the evaporation of most volatile solvent THF (Fig. S7a inset, ESI†). Furthermore, cryo-transmission electron microscopy (cryo-TEM) images show massive vesicles in the reaction mixture (Fig. S7, ESI†). These vesicles are in two states: a few vesicle-aggregates (Fig. S7a, marked by red arrows, ESI†) and separated vesicles under attaching with others (Fig. S7b, ESI†). These phenomena imply that the Al^3+^-based oligomers/PEO-*b*-PMMA composite vesicles are formed during the evaporation of THF, and these vesicles further attach to each other into big aggregates. It clearly reveals the important intermediate status during the formation process of the resultant as-made Al^3+^-based gel/PEO-*b*-PMMA composite microspheres.

**Scheme 1:**
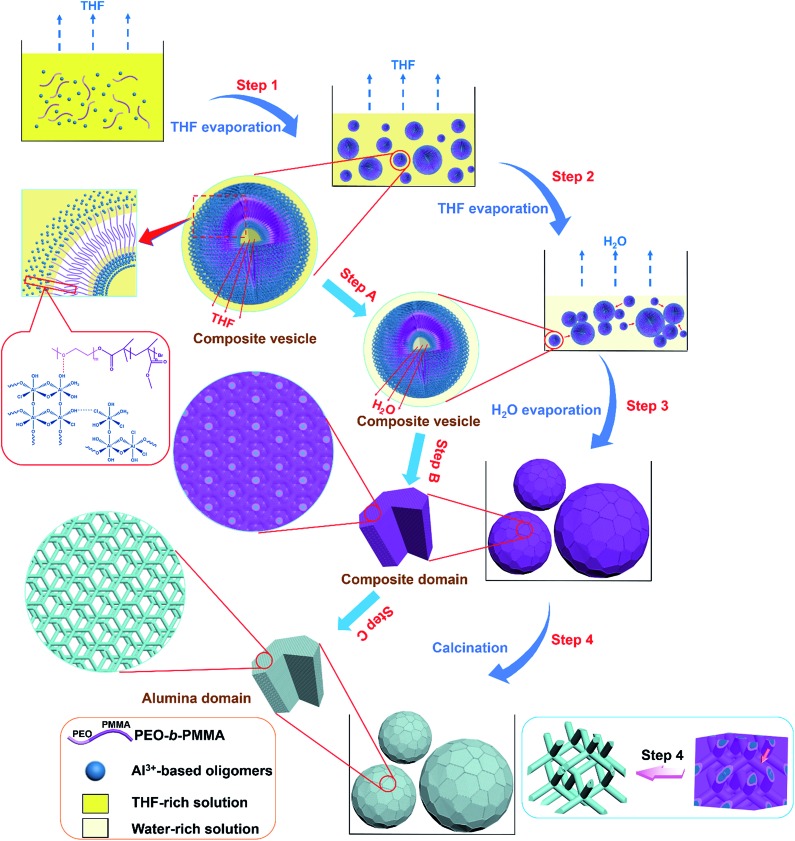
The formation process of ordered mesoporous γ-alumina microspheres with shifted double-diamond networks *via* a solvent evaporation induced vesicle-aggregation-assembly approach. Step 1: the formation of tiny Al^3+^-based oligomers/PEO-*b*-PMMA composite vesicles with PMMA segments as the hydrophobic interlayer and Al^3+^-based oligomer-associated PEO segments as the hydrophilic inner and outer walls caused by THF evaporation induced self-assembly. Step 2: the aggregation of composite vesicles into big composite vesicle-aggregates on the vesicle–solution interface driven by the ever-increasing concentration of composite vesicles and the requirement of reduction of interface energy with evaporation of remaining THF. Step 3: the transformation from composite vesicle-aggregates into as-made Al^3+^-based gel/PEO-*b*-PMMA composite microspheres with a double-diamond mesostructure caused by water evaporation induced microphase separation. Step 4: the formation of mesoporous alumina microspheres with a shifted double-diamond mesostructure owing to the decomposition of the template PEO-*b*-PMMA after calcination.

Based on the above results, we propose that the highly ordered mesoporous γ-alumina microspheres with shifted double-diamond networks are formed through a solvent evaporation induced vesicle-aggregation-assembly process (Scheme 1). In the first stage, the water-insoluble amphiphilic diblock copolymer PEO-*b*-PMMA can be dissolved well in a strong acidic solution with a high THF/H_2_O volume ratio due to the good solubility in THF. As the volatile solvent THF evaporates, the long hydrophobic PMMA segments of the template PEO-*b*-PMMA tend to aggregate to form hydrophobic domains, while the hydrophilic PEO segments are retained in the solution. In order to reduce surface tension, the polymer molecules self-assemble into vesicles at a very early stage, with PEO segments as inner and outer walls to dissolve in the solution and PMMA segments as hydrophobic domains to aggregate between the two walls. On the other hand, tiny Al^3+^-based oligomers in the solution controlled by the high acidity can interact with the PEO segments of the diblock copolymer PEO-*b*-PMMA by hydrogen bonding, giving the tiny Al^3+^-based oligomers/PEO-*b*-PMMA composite vesicles with different sizes (Scheme 1, Step 1). With continuous loss of THF in the inner solution of composite vesicles, the diameter of these vesicles becomes smaller gradually (Scheme 1, Step A). Meanwhile, the overall concentration of Al^3+^-based oligomers/PEO-*b*-PMMA composite vesicles increases as THF evaporates, driving the contact and attachment of vesicles to form big aggregates on the vesicle–solution interface to reduce interface energy (Scheme 1, Step 2). With continuous evaporation of solvent water in the later stage, massive HCl molecules are removed simultaneously, resulting in the gelation of Al^3+^-based oligomers. Meanwhile, as the water molecules left in hydrophilic domains are gradually removed, the reassembly-triggered mesophase-transformation (also referred to as microphase separation) of the composite vesicles occurs due to the increase in the volume ratio between hydrophobic PMMA domains and hydrophilic Al^3+^-based oligomers/PEO domains, resulting in the formation of the Al^3+^-based gel/PEO-*b*-PMMA composite domains with an inverse double-diamond mesostructure and single crystal-like morphology (Scheme 1, Step B). Therefore, the composite vesicle-aggregates transform into as-made Al^3+^-based gel/PEO-*b*-PMMA composite microspheres (Scheme 1, Step 3). In the subsequent calcination process, the two interpenetrating but disconnected alumina networks shift due to the decomposition of the template PEO-*b*-PMMA (Scheme 1, Step C). However, owing to the unique interlocking property of the bi-continuous cubic mesostructure, the collapse of the formed alumina microspheres is effectively avoided, only accompanied by a change of the structural symmetry from double diamond to shifted double diamond (single diamond) (Scheme 1, Step 4). Furthermore, the amorphous frameworks can be transformed into the crystallized frameworks composed of γ-Al_2_O_3_ nanocrystals without the destruction of the formed ordered mesostructure as the temperature is increased. This should be attributed to the maximum release of internal stress due to the unique rod-like alumina frameworks during the rearrangement of the atoms. Therefore, the mesoporous γ-Al_2_O_3_ microspheres with a shifted double-diamond mesostructure result from a complex process, mainly including the formation and aggregation of composite vesicles, the microphase separation between the hydrophobic domain and hydrophilic domain, and the shifting of two individual networks. In particular, the transformation of the mesophase from composite vesicle-aggregates to bicontinuous mesostructural composite microspheres is mainly attributed to the ratio change of the hydrophobic domain and hydrophilic domain as the reaction solvents evaporate.

**Fig. 5:**
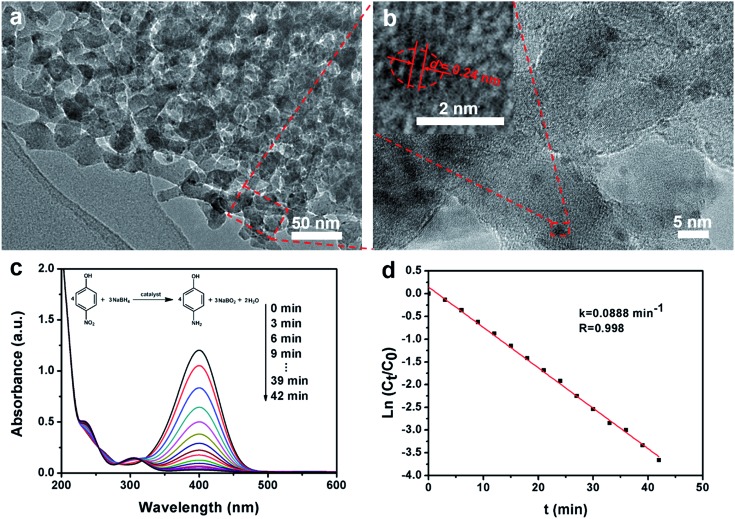
(a) The TEM image and (b) HRTEM image of Au/mesoporous γ-alumina composites after loading of Au nanoparticles. The inset in (b) shows a magnified TEM image of one Au nanoparticle. (c) UV-vis absorption spectra recorded during the catalytic reduction of 4-nitrophenol by sodium borohydride at 25 °C with 3 min intervals. (d) The relationship between ln(*C*_*t*_/*C*_0_) and reaction time (*t*), wherein the ratios of 4-nitrophenol concentration (*C*_*t*_ at time *t*) to its initial value *C*_0_ (*t* = 0) are directly given by the relative intensity of the respective absorbance *A*_*t*_/*A*_0_.

The highly ordered mesoporous γ-alumina obtained after calcination at 900 °C in air was employed as a support of Au nanoparticles for the catalytic application. TEM and STEM images show that the frameworks of ordered mesoporous γ-alumina are well retained after the loading of Au nanoparticles (Fig. 5a and S8a, ESI†). On the other hand, the uniform distribution of tiny Au nanoparticles (≤2 nm) is confirmed by the elemental mapping image and the HRTEM image (Fig. S8d, ESI† and Fig. 5b), and the content of Au is determined to be 1.51 wt% according to the EDX results (Fig. S8e, ESI†). The HRTEM image of one Au nanoparticle shows the lattice fringes with a spacing of ∼0.24 nm (Fig. 5b inset), which corresponds to the *d*_111_ of single-crystalline Au, further confirming that the Au nanoparticles are successfully synthesized and loaded on the columnar γ-alumina frameworks by the post-impregnation method. In the catalytic reduction process, the mixed solution of 4-nitrophenol and sodium borohydride firstly shows a strong absorption peak at 400 nm (Fig. 5c), which reflects the formation of 4-nitrophenolate ions.^51^ After the addition of the Au/mesoporous γ-alumina composite catalysts, the absorption peak at 400 nm decreases with time rapidly, and a new absorption peak appears and develops at 305 nm simultaneously, corresponding to the reduction of 4-nitrophenol to 4-aminophenol. The values of ln(*C*_*t*_/*C*_0_) *versus* the reaction time (*t*) show a good linear fitting and a kinetic constant *k* of 0.0888 min^–1^ (Fig. 5d), which is much higher than that of high Au-loaded mesoporous silica composites reported previously.^51^ The enhanced catalytic performance may stem from smaller Au nanoparticles, larger mesopores and better accessibility.

## Conclusions

In summary, a novel vesicle-aggregation-assembly approach induced by solvent evaporation has been demonstrated to synthesize highly ordered mesoporous γ-alumina microspheres with a unique shifted double-diamond mesostructure by using block copolymer PEO-*b*-PMMA as a soft template and aluminum isopropoxide as a precursor in an acidic THF and water binary solvent. During evaporation of THF and subsequent water in the mixed solution, a complex co-assembly process, including formation of composite vesicles, aggregation between these vesicles and microphase separation of composite vesicle-aggregates, leads to the formation of as-made Al^3+^-based gel/PEO-*b*-PMMA composite microspheres with an inverse double-diamond mesostructure (*Pn*3[combining macron]*m*). Moreover, after calcination at 900 °C in air to remove the template and crystallization of the alumina frameworks, a change of structural symmetry occurs from *Pn*3[combining macron]*m* to *Fd*3[combining macron]*m*, which results from the shifting of two intertwined but disconnected alumina networks. The obtained alumina microspheres are composed of columnar and crystallized γ-alumina networks (nanocrystal size ∼ 15 nm), and these networks create porous space with ultra-large mesopores (∼72.8 nm). Furthermore, the γ-alumina microspheres exhibit thermal stability as high as 900 °C. Finally, the novel mesoporous γ-alumina material can be used as a support of Au nanoparticles in the catalytic reduction of 4-nitrophenol with sodium borohydride. This work may pave a promising way in the designed synthesis of novel mesoporous materials with unique mesostructures and morphologies.

## Conflicts of interest

There are no conflicts to declare.

## Supplementary Material

Supplementary information
