# Supplementary material for: A vesicle-aggregation-assembly approach to highly ordered mesoporous γ-alumina microspheres with shifted double-diamond networks
Source: Chem Sci. 2018 Aug 17;9(39):7705–14. doi: 10.1039/c8sc02967a (PMC6182608; doi:10.1039/c8sc02967a)
Supplement: Supplementary file 1 [file SC-009-C8SC02967A-s001.pdf]

Electronic Supplementary Material (ESI) for Chemical Science.
This journal is © The Royal Society of Chemistry 2018

RSCPublishing

Supplementary Information

A vesicle-aggregation-assembly approach to highly ordered mesoporous γ-alumina microspheres with shifted double-diamond networks

Yang Liu,[a] Wei Teng,[b] Gang Chen,[c] Zaiwang Zhao,[a] Wei Zhang,[a] Biao Kong,[a] Wael N. Hozzein,[d] Areej Abdulkareem Al-Khalaf,[e] Yonghui Deng[af] and Dongyuan Zhao*[a]

[a] Department of Chemistry, Laboratory of Advanced Materials, Shanghai Key Laboratory of Molecular Catalysis and Innovative Materials, and iChEM, Fudan University, Shanghai 200433, P. R. China

[b] State Key Laboratory for Pollution Control, School of Environmental Science and Engineering, Tongji University, Shanghai 200092, P. R. China

[c] School of Physical Science and Technology, ShanghaiTech University, Shanghai 201210, P. R. China

[d] Bioproducts Research Chair, Zoology Department, College of Science, King Saud University, Riyadh 11451, Saudi Arabia. Botany and Microbiology Department, Faculty of Science, Beni-Suef University, Beni-Suef, Egypt

[e] Biology Department, College of Sciences, Princess Nourah bint Abdulrahman University, Riyadh, Saudi Arabia

[f] State Key Laboratory of Transducer Technology, Shanghai Institute of Microsystem and Information Technology, Chinese Academy of Sciences, Shanghai 200050, P. R. China

Email: dyzhao@fudan.edu.cn

Experimental Section

Chemicals.

Monomethoxy poly(ethylene oxide) (Mw 5000 g/mol) was purchased from Aldrich. 2-Bromoisobutyryl bromide (98%) was obtained from Acros. N,N,N',N'',N''-pentamethyldiethylenetriamine (98%) was supplied by J&K China Chemical Ltd. Methyl methacrylate (CP), copper bromide (CP), aluminum isopropoxide (CP), 4-nitrophenol (AR), sodium borohydride (AR), $HAuCl_4 \cdot 4H_2O$ (AR), triethylamine (AR), ethylenediamine (AR), hydrochloric acid (37 wt%), tetrahydrofuran (AR), dichloromethane (AR), anhydrous diethyl ether (AR), acetic acid (AR), alcohol (AR), petroleum ether (60-90 °C) and neutral alumina (FCP, 200-300 mesh) were purchased from Sinopharm Chemical Reagent Co., Ltd. Monomer methyl methacrylate was purified by neutral alumina column to remove the polymerization inhibitor before used. CuBr was washed with acetic acid and alcohol for three times, respectively, and then dried under vacuum. Other chemicals were used as received without any further treatment. Millipore water was used in all experiments.

Synthesis of PEO_{113}-Br.

The macroinitiator monomethoxy poly(ethylene oxide)$_{113}$ with a bromic terminal group (PEO_{113}-Br) was synthesized by acylation of monomethoxy poly(ethylene oxide) (PEO_{113}) with 2-bromoisobutyryl bromide. Typically, PEO_{113} (40 g) and triethylamine (7.3 mL) were mixed in dichloromethane (100 mL) under stirring. After 2-bromoisobutyryl bromide (3.24 mL) was quickly added into the above solution, the resultant solution in the sealed flask was kept stirring in an oil bath under 30 °C for 48 h. Then the solution was washed with water (80 mL) for two times, concentrated, and dripped into a large amount of anhydrous diethyl ether to precipitate PEO_{113}-Br. The product PEO_{113}-Br was collected by filtration and dried under vacuum.

Synthesis of PEO_{113}-*b*-$PMMA_{335}$.

Diblock copolymer poly(ethylene oxide)$_{113}$-*block*-poly(methyl methacrylate)$_{335}$ (PEO_{113}-*b*-$PMMA_{335}$) was prepared by an atom transfer radical polymerization (ATRP) method. Firstly, PEO_{113}-Br (2.5 g) and N,N,N',N'',N''-pentamethyldiethylenetriamine (230 μL) were added into a mixed solution containing tetrahydrofuran (THF) (30 mL) and methyl methacrylate (MMA) (30 mL). Then the reaction solution in the sealed bottle was bubbled with argon for 30 min before copper bromide (75 mg) was added under argon atmosphere quickly. After bubbling for another 30 min, the resealed bottle was placed in an oil bath at 60 °C for 3 h under stirring. Afterwards, the resultant viscous solution was quickly taken out from the oil bath, cooled down to room temperature, diluted with dichloromethane and exposed to the air with stirring before it was filtered through a neutral alumina column to remove the copper complex using dichloromethane as an eluent. After the filtrate was concentrated under vacuum, PEO_{113}-*b*-$PMMA_{335}$ was precipitated in petroleum ether, collected by filtration and dried under vacuum.

Loading of Au Nanoparticles.

Au nanoparticles were loaded onto the ordered mesoporous γ-alumina *via* an impregnation-reduction approach. Firstly, ethylenediamine (115 μL) was added into 0.10 g/mL $HAuCl_4 \cdot 4H_2O$ aqueous solution (2.0 mL) under stirring until a transparent orange solution was formed. Then alcohol (16 mL) was added to precipitate the $Au(en)_2Cl_3$ complex, and the complex was separated by centrifugation, washed with alcohol for two times, and dried under vacuum at 40 °C for 1 day. $Au(en)_2Cl_3$ (10 mg) was dissolved in water (5.0 mL), and the pH value was modified to 10 with NaOH aqueous solution (5.0 wt%). The mesoporous γ-alumina obtained after calcination at 900 °C in air (60 mg) was dispersed in the above solution under stirring for 1 h. The sample was separated by centrifugation, washed with water quickly, and dried under vacuum at 40 °C for 2 days. The sample was reduced with H_2/Ar (5.0 %) stream at 150 °C for 1 h, resulting in the Au/mesoporous γ-alumina composites.

Catalytic Reduction of 4-nitrophenol.

The reduction of 4-nitrophenol was carried out in a quartz cuvette and monitored by using a UV-vis spectroscopy at 25 °C. Typically, 0.2 mM 4-nitrophenol aqueous solution (1.0 mL) was mixed with 0.1 M sodium borohydride aqueous solution (2.0 mL). Subsequently, 200 μL of Au/mesoporous γ-alumina composites aqueous solution (0.2 mg/mL) was added, and the mixed solution was quickly subjected to UV-vis measurements for monitoring the absorption changes during the reaction in the range of 200-600 nm at 3 min internals.

Measurements and Characterization.

^{1}H NMR spectrum was recorded on a Varian Mercury Plus 400 MHz NMR spectrometer with tetramethylsilane (TMS) as the internal reference. The molecular weight distribution of the diblock copolymer PEO-*b*-PMMA was estimated by an Agilent 1100 gel permeation chromatography (GPC) system using THF as the eluent against polystyrene standards with a RID detector. Field-emission scanning electron microscopy (FESEM) images were taken by a Hitachi S4800 scanning electron microscope. Samples were directly dispersed onto conductive tapes attached on a sample holder for direct observation under vacuum. Transmission electron microscopy (TEM) experiments were conducted on a JEOL JEM-2100F microscope (Japan) operated at 200 kV. The samples for TEM measurements were suspended in ethanol and dropped onto Cu grids. To load the as-made Al^{3+}-based gel/PEO_{113}-*b*-$PMMA_{335}$ composites, the Cu grids were mixed with composites and then fetched out for the tests. To obtain more detailed information during the solvent evaporation induced vesicle-aggregation-assembly process, cryo-transmission electron microscopy (cryo-TEM) images were taken on a FEI Tecnai G20 microscopy (Netherlands) operated at 200 kV, and the samples for cryo-TEM tests were made on a Vitrobot specimen-making machine by quickly freezing the reactant solution-loaded Cu girds with liquid nitrogen. Thermogravimetric analysis (TGA) experiments were conducted on a PerkinElmer TGA 4000 analyzer. X-ray diffraction (XRD) measurements were performed on a Bruker D8 Advance X-ray diffractometer using a CuKα radiation source (λ = 1.5406 Å).

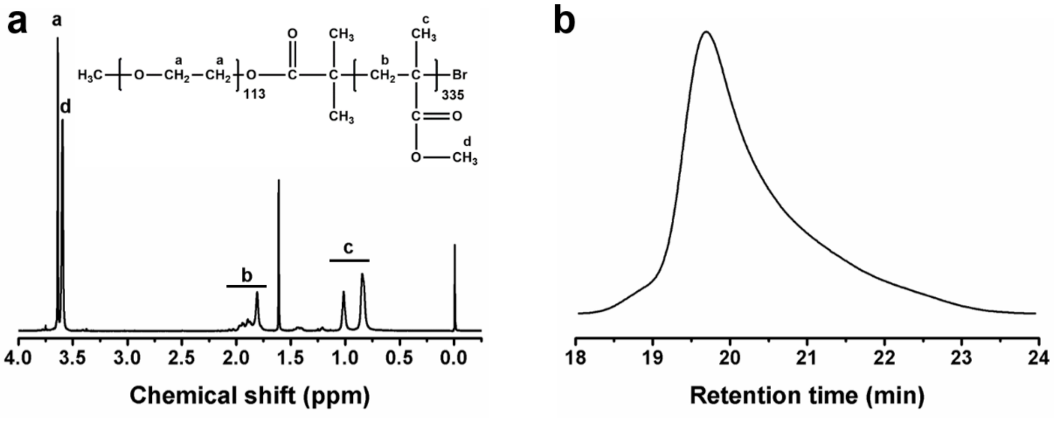

Fig. S1 (a) The ^{1}H NMR spectrum (400 MHz, $CDCl_3$) and (b) GPC plot of the block copolymer PEO_{113}-*b*-$PMMA_{335}$. The molecular weight of the PMMA block (M_{PMMA}) was determined by the formula:

$$\frac{M_{PEO}}{M_{PMMA}} = \frac{(I_{a+d} - I_c) \times 44/4}{I_c \times 100/3}$$

so the number of repetitive units calculated from M_{PMMA} is about 335. The polydispersity index (PDI) is 1.31, according to the GPC tests.

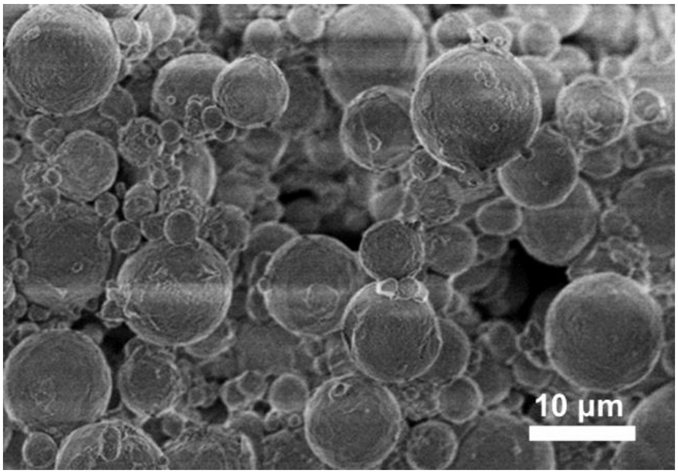

Fig. S2 The FESEM image of as-made Al^{3+}-based gel/PEO-*b*-PMMA composite microspheres prepared by the vesicle-aggregation-assembly approach.

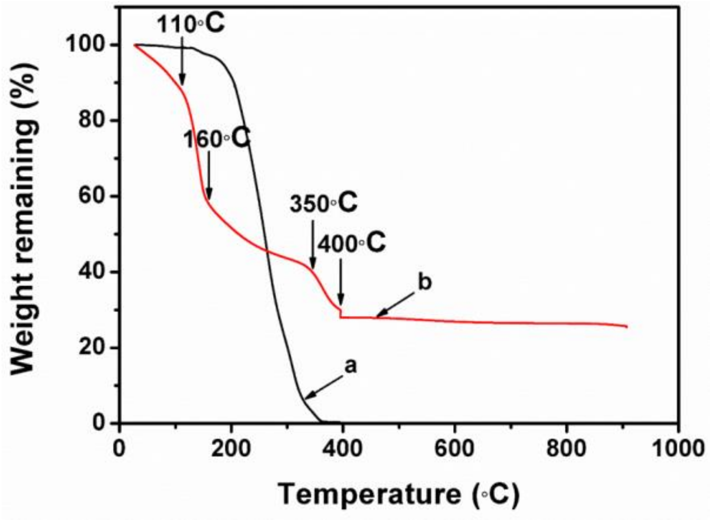

Fig. S3 TGA curves of (a) the diblock copolymer PEO-*b*-PMMA and (b) as-made Al^{3+}-based gel/PEO-*b*-PMMA composites following the same ramping rate and holding time as that of mesoporous alumina obtained after calcination at 900 °C in air.

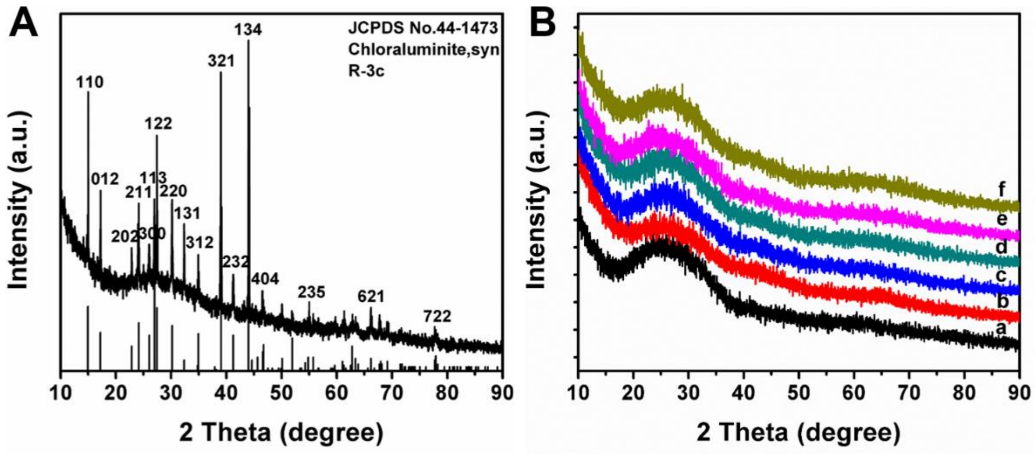

Fig. S4 Wide-angle XRD patterns of the intermediate products obtained after the as-made Al^{3+}-based gel/PEO-*b*-PMMA composites were calcined from 25 °C to (A) 100 °C, (B) (b) 150 °C, (c) 200 °C, (d) 250 °C, (e) 300 °C and (f) 350 °C with a ramp rate of 1 °C/min in air. The wide-angle XRD pattern shown in B(a) is corresponded to the glass substrate used as a sample support for a reference.

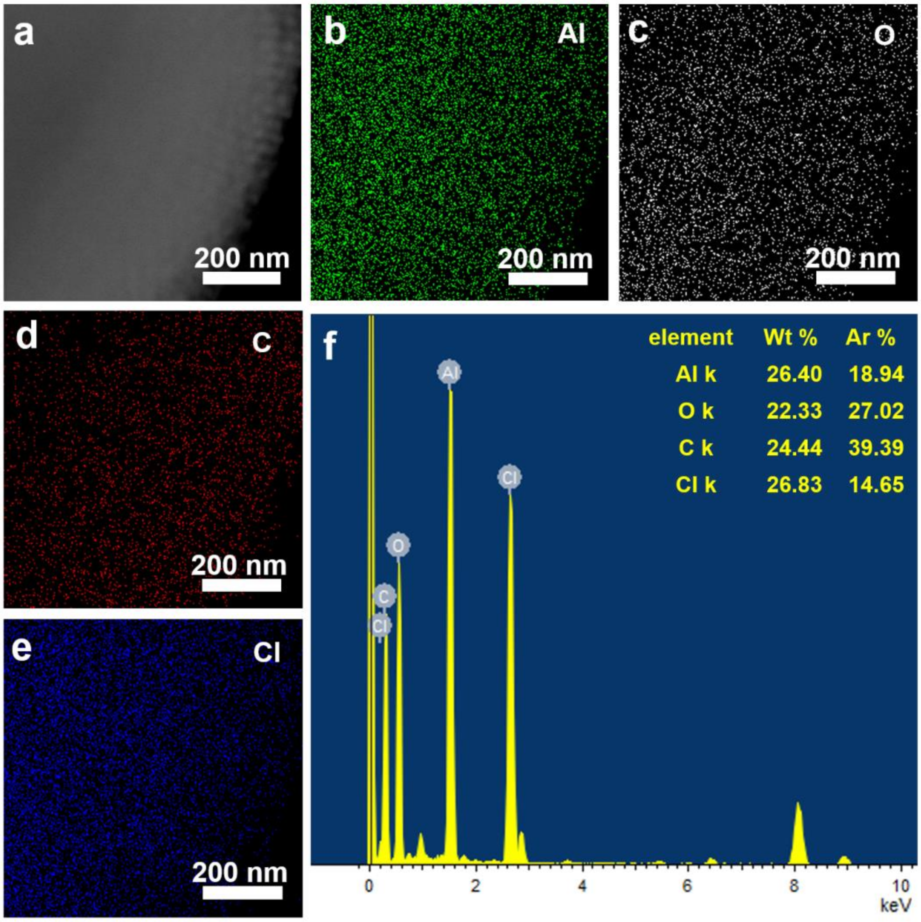

Fig. S5 (a) The dark-field scanning transmission electron microscopy (STEM) image, (b-e) the corresponding element mapping images (element Al, O, C and Cl) and (f) the energy dispersive X-ray (EDX) spectrum of the as-made Al^{3+}-based gel/PEO-*b*-PMMA composites prepared by the vesicle-aggregation-assembly approach. The table inserted in (f) shows the chemical composition of the selected area.

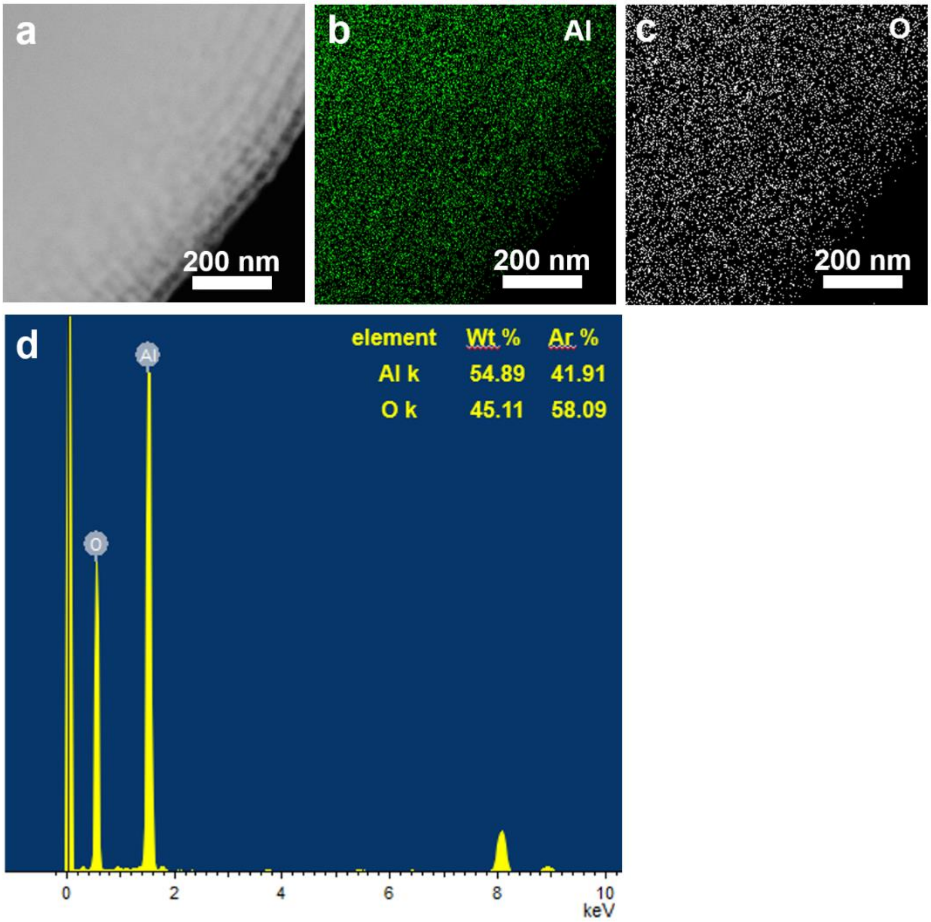

Fig. S6 (a) The STEM image, (b, c) the corresponding element mapping images (element Al and O) and (d) the EDX spectrum of the ordered mesoporous alumina microspheres obtained after calcination at 900 °C in air. The table inserted in (d) shows the chemical composition of the selected area.

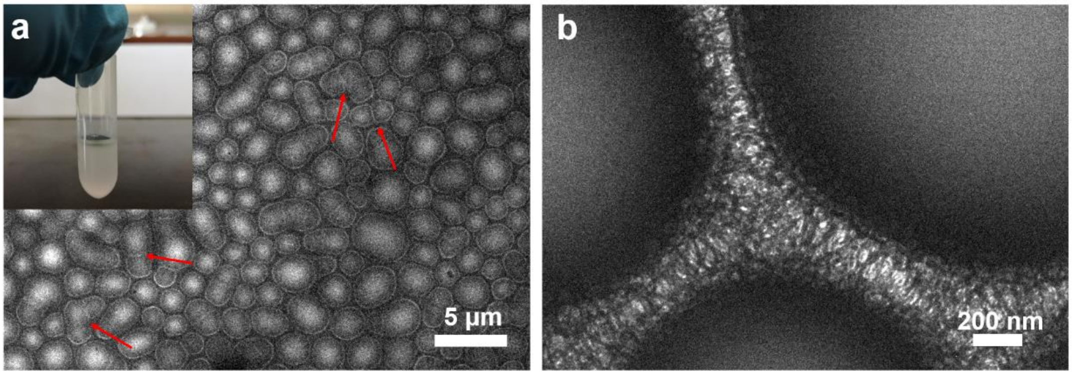

Fig. S7 Cryo-TEM images of the intermediate reaction solution taken from (a) a large area and (b) a local area after the evaporation of the reaction solvents at room temperature for 2 h. The inset in (a) shows the optical photograph of the intermediate reaction solution.

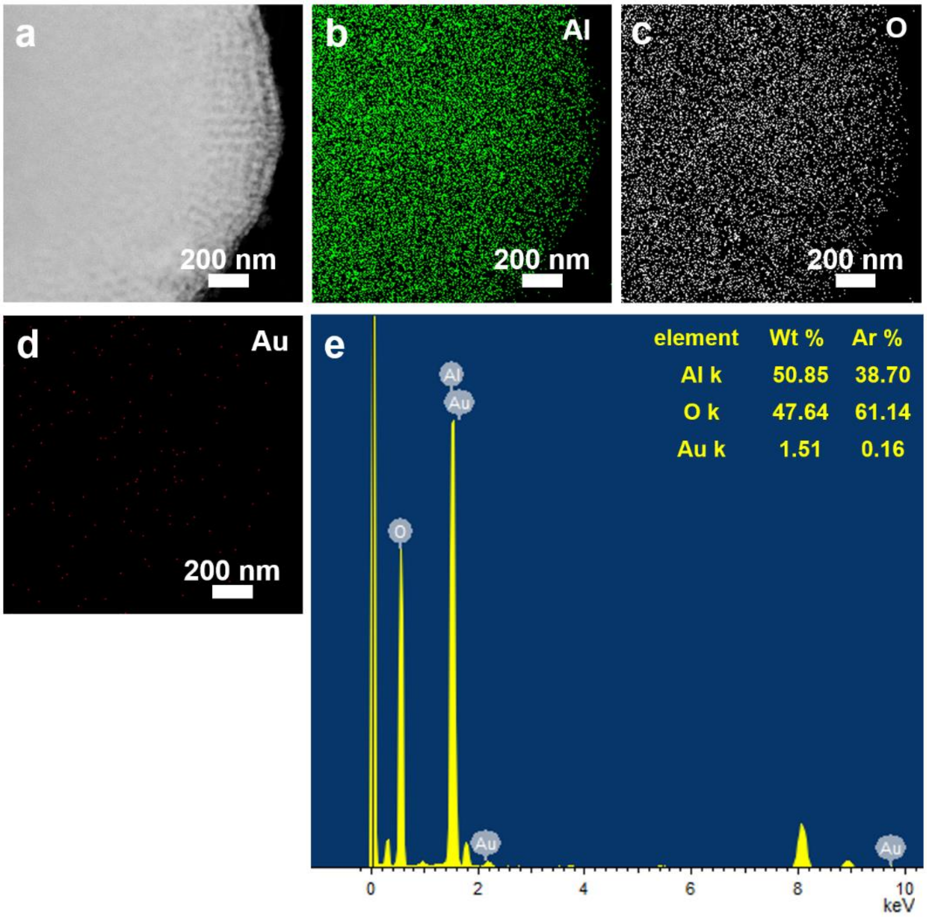

Fig. S8 (a) The STEM image, (b-d) the corresponding element mapping images (element Al, O and Au) and (e) the EDX spectrum of the Au/mesoporous γ-alumina composites after loading Au nanoparticles by an impregnation-reduction approach. The table inserted in (e) shows the chemical composition of the selected area.
